# Minocycline Ameliorates *Staphylococcus aureus*-Induced Neuroinflammation and Anxiety-like Behaviors by Regulating the TLR2 and STAT3 Pathways in Microglia

**DOI:** 10.3390/brainsci15020128

**Published:** 2025-01-28

**Authors:** Jiao Zou, Junwei Gao, Weilong Shang, Xiaotang Fan

**Affiliations:** 1 Department of Military Cognitive Psychology, School of Psychology, Third Military Medical University (Army Medical University), Chongqing 400038, China; zoujiao89@tmmu.edu.cn (J.Z.); junwei_gao@tmmu.edu.cn (J.G.); 2 Key Laboratory of Microbial Engineering Under the Educational Committee in Chongqing, Department of Microbiology, College of Basic Medical Sciences, Third Military Medical University (Army Medical University), Chongqing 400038, China; shangwl@tmmu.edu.cn

**Keywords:** anxiety, minocycline, neuroinflammation, microglia, TLR2, STAT3

## Abstract

**Background:** Anxiety disorders are the most common mental illnesses. *S. aureus* is a Gram-positive opportunistic pathogen most commonly associated with anxiety-like behaviors. Minocycline ameliorates Gram-negative bacterial LPS-induced anxiety-like behaviors by suppressing microglia activation. However, the effects of minocycline on anxiety-like behaviors caused by *S. aureus* infections have received little attention. In this study, we aimed to investigate the molecular mechanism and effect of minocycline on anxiety-like behaviors caused by *S. aureus* infection. **Methods:** BV2 and N9 microglial cells were treated in vitro. The effects of minocycline on lipoteichoic acid (LTA)-stimulated inflammatory responses, STAT3 activation, and GLS1 expression were assessed using Western blotting, and cytokine secretion was determined using an ELISA. A mouse model was used to evaluate the capacity of minocycline to ameliorate anxiety-like behaviors caused by *S. aureus* infection. **Results:** We found that ≥100 μmol/L of minocycline remarkably attenuated LTA-induced TLR2 signaling pathway activation and proinflammatory cytokine expression in microglial cells. Minocycline prevented LTA-stimulated STAT3 activation and GLS1 expression in vitro. LTA-induced TLR2, TNF-α, IL-6, and GLS1 expression was markedly reduced by the inhibition of STAT3 phosphorylation. Mice were pretreated with 50 mg/kg of minocycline, significantly attenuating microglial activation and neuroinflammation. Minocycline also effectively alleviated the anxiety-like behaviors induced by *S. aureus* infection. **Conclusions:** Our findings indicate that minocycline alleviates *S. aureus* infection-induced anxiety-like behaviors by suppressing microglia activation.

## 1. Introduction

Anxiety disorders affect 7.3% to 28.0% of the population [1,2]. A complex interaction among social, psychological, and biological factors could contribute to anxiety disorders. Microbial infection-induced neuroinflammation often causes anxiety disorders [3,4]. A myriad of microorganisms are closely associated with human health, disease, and interactions with the central nervous system (CNS) [5,6]. In addition to affecting the gut microbiota, bacterial or viral infections can also induce neuroinflammation [7,8,9,10].

Located in the CNS, microglial cells are resident immune cells that monitor the microenvironment around them [11]. Microglia express all recognized toll-like receptors (TLRs), which are able to identify pathogen- or damage-associated molecular patterns (PAMPs, DAMPs) [12]. Numerous studies have shown that activated microglia create proinflammatory cytokines, which contribute significantly to neuroinflammation [12,13]. Clinical investigations have revealed that anxiety- and depressive-like behaviors are related to neuroinflammation caused by microglia overstimulation [12]. Lipoteichoic acid (LTA) is an important Gram-positive pathogen antigen that has been shown to promote microglia activation in a TLR2-dependent manner [14,15,16]. The blood–brain barrier (BBB) can be disrupted by LTA, which stimulates microglia to release proinflammatory cytokines and leads to neuroinflammation and even anxiety-like behavior [13].

Due to its high lipid solubility, minocycline can readily cross the BBB [17]. According to previous studies, minocycline exhibits anti-inflammatory, microglia inhibitory, and neuroprotective effects in the CNS [4,17]. Minocycline promotes neuroprotection by modulating the PI3/Akt/GSK or BDNF/CREB signaling pathways [18,19]. Gram-negative bacterial lipopolysaccharide (LPS) penetrates the brain via a lipoprotein-mediated transfer process to induce neuroinflammation and anxiety-like behavior [20,21,22]. Previous studies have shown that minocycline administration attenuated LPS-induced anxiety-like behaviors in a mouse model [23,24]. LPS-activated TLR4 signaling promotes the phosphorylation of the signal transducer and activator of transcription 3 (STAT3) and triggers the release of proinflammatory cytokines in microglia [25]. Long et al. recently reported that minocycline offers therapeutic benefits in terms of reducing microglia activation in neuroinflammatory diseases by attenuating the LPS-activated JAK2-STAT3 inflammatory signaling pathway [26]. STAT3 targets the glutaminase (GLS) promoter to enhance GLS expression [27]. GLS catalyzes the hydrolysis of glutamine (Gln) to produce glutamate (Glu) [27]. Glutamate, released by activated microglia, stimulates the glutamate receptors on microglia to promote the release of cytokines [28]. Additionally, glutamate is the major excitatory neurotransmitter in the CNS and is linked to emotion regulation processing [29].

*Staphylococcus aureus* (*S. aureus*) is a major Gram-positive pathogen. In addition to infectious diseases, *S. aureus* can cause a range of other clinical manifestations [30]. Our prior research showed that *S. aureus* infection in BALB/C mice could induce anxiety-like behaviors, which were associated with microglia activation and neuroinflammation [31]. During *S. aureus* infection, the recognition of *S. aureus* peptidoglycan (PGN), lipoproteins, and LTA by TLR2 triggers the secretion of proinflammatory cytokines [32]. However, minocycline’s effects in terms of ameliorating *S. aureus* infection-induced TLR2-dependent microglia activation and anxiety-like behaviors are still unclear. The present study aimed to determine the molecular mechanism by which minocycline suppresses *S. aureus* infection-induced microglia activation. The effects of minocycline on *S. aureus* infection-induced anxiety-like behaviors were also examined.

## 2. Materials and Methods

### 2.1. Bacterial Strain and Growth Condition

The *S. aureus* USA300 strain (GenBank accession no. CP000255.1) was cultured with brain heart infusion (BHI) medium (Oxoid, Starstoke, UK) at 37 °C.

### 2.2. Mice

Female mice exhibited higher levels of estrogen. To eliminate any potential confusion regarding the effects of estrogen in female mice, we used male mice in our in vivo experiments. Male BALB/C mice weighing 20–24 g were employed in this study. The animals were housed under a light cycle (lights on at 8:00 AM–8:00 PM) in a plastic cage with five individuals per cage and ad libitum access to tap water and commercial feed.

### 2.3. Chemicals and Antibodies

LTA (extract from *S. aureus*) and minocycline were purchased from the Sigma-Aldrich Corporation (St. Louis, MO, USA). The STAT3 phosphorylation (at Y705 and S727) inhibitor Stattic was purchased from MCE (Princeton, NJ, USA). The CCK-8 assay kit was purchased from Dojindo (Kumamoto, Japan). The BCA kit was purchased from Beyotime Biotechnology (Shanghai, China). Rabbit monoclonal anti-TLR2, IL-6, TNF-α, and GLS1 antibodies were purchased from Abcam (Cambridge, UK). The total and phosphorylated STAT3 antibodies were obtained from Cell Signaling Technology (Danvers, MA, USA). The GAPDH and secondary antibodies were supplied by the Zhongshan Company (Beijing, China). Rabbit anti-IBA1 was obtained from Wako (Tokyo, Japan). The rabbit anti-Cy3 was obtained from Invitrogen (Carlsbad, California, USA). DAPI was purchased from Roche (Basel, Switzerland). Mouse IL-10, TNF-α, and IL-6 ELISA kits were obtained using a commercial ELISA kit (Minneapolis, MN, USA).

### 2.4. Cell Culture and CCK8 Assay

The murine microglial cell lines BV2 and N9 were supplied by the Wuhan Servicebio Technology Co., Ltd (Wuhan, China). The BV2 immortalized murine microglial cell line was generated by infecting primary microglial cell cultures with a retrovirus carrying the v-raf/v-myc oncogene. In contrast, the N9 cell line was generated by immortalizing E13 mouse embryonic brain cultures with the 3RV retrovirus, which carried an activated v-myc oncogene [33,34]. The microglial cell lines BV2 and N9 were maintained in DMEM supplemented with 10% fetal bovine serum at 37 °C with 5% CO_2_. For the experiments, the cells were seeded on a 96-well plate, followed by treatment with different doses (50–200 μmol/L) of minocycline (catalog no. M9511, Sigma-Aldrich, USA) for 0.5 h. After being pretreated with minocycline, the cells were treated with 5 μg/mL of LTA (catalog no. L2515, Sigma-Aldrich, USA) for 24 h. Finally, the cell viability was assessed using the CCK-8 assay kit (catalog no. CK13, Dojindo, Japan). Ten microliters of cell viability assay kit solution was added to each plate well, and the OD values at 450 nm were determined using a spectrophotometer.

### 2.5. Animal Experiment Design

**Figure 1:**
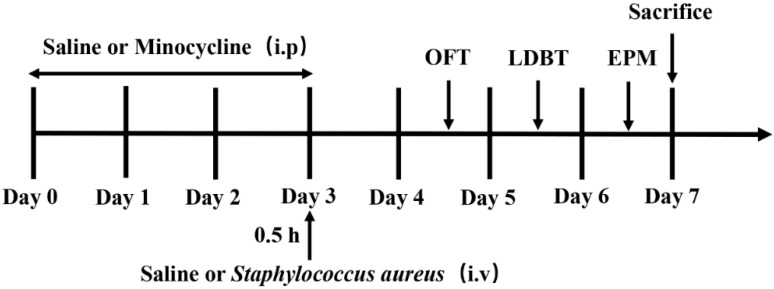
The design of the animal experiment.

We established an *S. aureus* infection mice model, as described previously [31]. The mice were intraperitoneally (i.p) injected with 100 μL saline or 50 mg/kg minocycline once daily for three days. At 0.5 h after the last dosage, the mice were intravenously (i.v) administered 100 μL saline or USA300 (1 × 10^5^ CFU). Four experimental groups of mice were established: (1) control, saline-treated group; (2) Mino, mice pretreated with minocycline and then challenged with the saline group; (3) USA300, mice pretreated with saline and then challenged with the USA300 group; (4) Mino + USA300, mice pretreated with minocycline and then challenged with the USA300 group. The experimental protocol is illustrated in Figure 1. All mouse body weights were measured at the same time every day, and the survival rates in the four experimental groups were noted.

### 2.6. Behavioral Testing

All behavioral tests were conducted between 9:00 AM and 5:00 PM. The elevated plus maze (EPM), light–dark box test (LDBT), and open field test (OFT) were carried out to assess the anxiety-like behavior of the mice. The specific procedures were implemented according to those used in our previous study [31]. For each mouse, the test equipment was cleaned with 75% ethanol between the tests.

#### 2.6.1. OFT

The mice were placed into the center of the open field (40 cm × 40 cm × 30 cm) and allowed to explore freely for 30 min. The movements of the mice were recorded for 30 min using a video camera secured to the top of the apparatus, and the total distance and time spent in the center were analyzed using Ethovision XT 11.0 (Noldus, Wageningen, Netherlands).

#### 2.6.2. LDBT

Each mouse was placed gently in the center of the light compartment (~400 l×), facing the aperture connected with the dark compartment, and allowed to move freely for 10 min. The amount of time spent on the dark side and the total number of transitions were recorded using Ethovision XT 11.0 (Noldus).

#### 2.6.3. EPM

The EPM was structured with two open arms positioned opposite to one another and two closed arms, also positioned opposite to one another (30 cm × 6 cm × 15 cm), extending from a central area (6 cm × 6 cm). Each mouse was gently placed in the center area facing an open arm and allowed to explore freely for 10 min. The percentage of time spent in the open arms and the percentage of open arm entries were analyzed.

### 2.7. Cytokine ELISA

Cells were seeded into 6-well plates with 0.5 × 10^6^ cells/well, followed by treatment with various concentrations of Stattic (catalog no. HY-13818, MCE, USA) or minocycline for 30 min; they were then stimulated with LTA 5 μg/mL for 5.5 h. The serum and culture supernatant were diluted according to the manufacturer’s instructions and assayed for interleukin (IL)-6 (catalog no. M6000B-1, R&D Systems, Minneapolis, MN, USA), IL-10 (catalog no. M1000B-1, R&D Systems, USA), and tumor necrosis factor (TNF)-α (catalog no. MTA00B-1, R&D Systems, Minneapolis, MN, USA) using a commercial ELISA kit.

### 2.8. Western Blot

After removing the culture media for each experimental groups, the cells were lysed with 200 μL RIPA Lysis Buffer (catalog no. P0013K, Beyotime, Shanghai, China) supplemented with 1% (*w*/*v*) phenylmethanesulfonyl fluoride (PMSF) (catalog no. ST506, Beyotime, China). The cell lysate was collected via centrifugation. The protein was extracted from fresh mouse mPFC tissues after the behavioral testing. The protein contents in the cell and mouse samples were measured using the BCA protein assay (catalog no. P0010S, Beyotime, China). Then, 50 μg protein samples were separated on 10% SDS-PAGE, and electrophoresis was applied at a constant pressure of 80 V for 120 min; the samples were then transferred to polyvinylidene fluoride (PVDF) membranes at 25 V for 35 min. Then, the membranes were blocked with phosphate-buffered saline (PBS) containing 0.1% Tween-20 (PBST) and 5% fat-free milk for 1 h at room temperature. Membranes were incubated overnight at 4 °C with primary antibodies against the following proteins: TLR2 (1:1000, catalog no. ab209216, Abcam, Cambridge, UK), TNF-α (1:1000, catalog no. ab183218, Abcam, UK), IL-6 (1:1000, catalog no. ab233706, Abcam, UK), STAT3 (1:1000, catalog no. 9139, CST, Massachusetts, USA), Phospho-Stat3 (Tyr705) (1:1000, catalog no. 9145, CST, USA), GLS1 (1:1000, catalog no. ab156876, Abcam, UK), and GAPDH (1:1000, catalog no. TA-08, Zhongshan, Beijing, China). The membrane was then washed three times with PBST, incubated with a secondary mouse (1:5000, catalog no. ZB-2305, Zhongshan, China) or rabbit (1:5000, catalog no. ZB-2301, Zhongshan, China) antibody for 1 h, and washed three times with PBST. The blots on the membrane were detected using ECL Western blot detection reagent kits. Finally, the expression of target proteins was measured using ImageJ, with GAPDH as the internal control for normalization. The Western blot experiment was conducted at least three times, and three points were used to plot the graphs.

### 2.9. Immunofluorescent Staining

After applying anesthesia with 10% sodium pentobarbital, each mouse was perfused transcranial with ice-cold PBS, followed by a fixative mixture of 4% paraformaldehyde (PFA) solution. The brains of the mice were placed in 4% PFA for 48 h. The dehydration of the brain was performed with 4% PFA solution with 30% sucrose. Coronal sections with a thickness of 30 μm were cut continuously with a cryostat. The sections were rinsed with PBS three times. After blocking the brain slices in blocking buffer for an hour at room temperature, they were incubated with the rabbit anti-IBA1 (1:500, catalog no. 019-19741, Wako, Tokyo, Japan) antibody overnight at 4 °C. Then, the slices were washed three times with PBS, and the secondary antibody, rabbit anti-Cy3 (1:500, catalog no. A10520, Invitrogen, Carlsbad, CA, USA), was added for 2 h. The sections were incubated with DAPI (1:40,000, catalog no. 10236276001, Roche, Basel, Switzerland) for approximately 1 min. Finally, images were acquired, and an analysis was performed. The numbers of IBA1-labeled microglial cells in 3–5 sections of mPFC were manually counted, and the counting area was determined according to the Mouse Brain in Stereotaxic Coordinates atlas. The statistical index was the average density (number/mm^2^).

### 2.10. Statistical Analysis

GraphPad Prism 9.0.0 software was used for the data analysis. Each experiment was conducted at least three times. The results were recorded as the mean ± SEM, and a *p* value of less than 0.05 was considered statistically significant. A repeated-measures ANOVA was used to assess the data on the mouse body weight, the time spent in the center in the OFT, and the total distance in the OFT. A one-way ANOVA and post hoc Tukey–Kramer’ multiple comparison test were used to evaluate the group differences.

## 3. Results

### 3.1. Minocycline Inhibits LTA-Induced Proinflammatory Cytokine Production in Microglia

**Figure 2:**
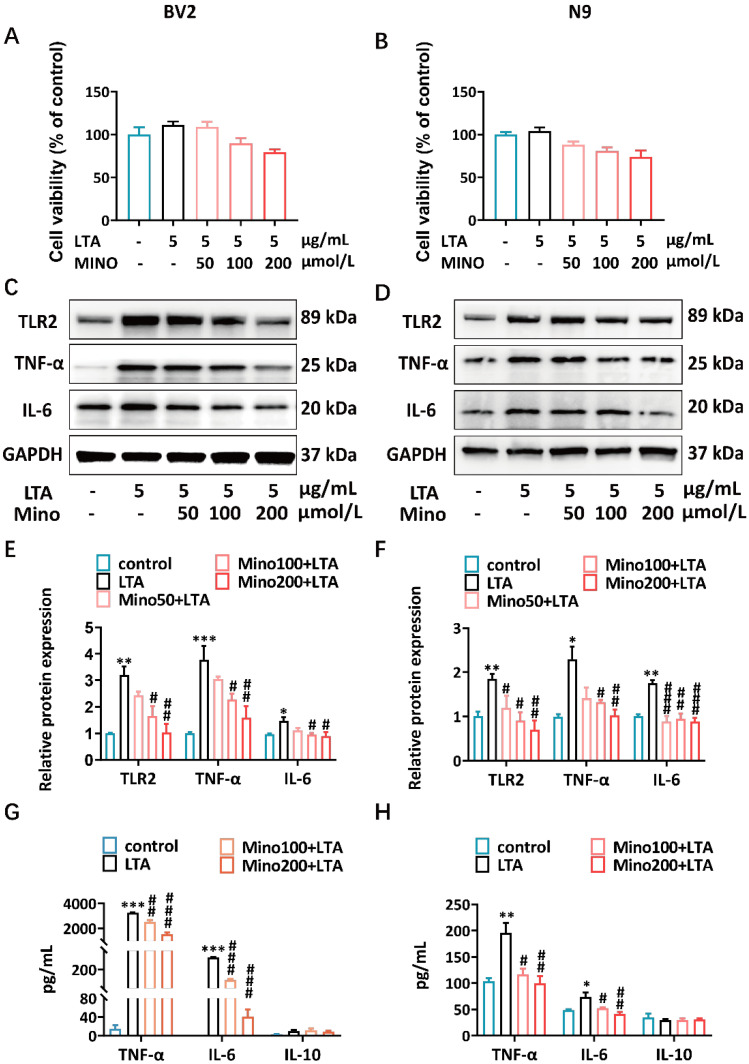
Effects of minocycline on inflammatory response in LTA-induced microglial cells. (**A**,**B**) The cell viability rates of BV2 and N9 cells. (**C**,**D**) The TLR2, TNF-α, and IL-6 protein expression levels in BV2 and N9 cells were analyzed via Western blot analysis. (**E**,**F**) The quantification of TLR2, TNF-α, and IL-6 protein expression in BV2 and N9 cells. The data were normalized according to GAPDH. (**G**,**H**) The levels of TNF-α, IL-6, and IL-10 secreted by BV2 and N9 cells were determined via ELISA. The mean ± SEM of three separate experiments is represented for all data that have error bars. Compared to the control group, * *p* < 0.05; ** *p* < 0.01; *** *p* < 0.001. Compared to the LTA group, ^#^ *p* < 0.05; ^##^ *p* < 0.01; ^###^ *p* < 0.001. Control, saline-treated group; LTA, cells pretreated with saline and then stimulated with LTA; Mino100 + LTA, cells pretreated with 100 μmol/L minocycline and then stimulated with LTA; Mino200 + LTA, cells pretreated with 200 μmol/L minocycline and then stimulated with LTA.

Previous work reported that BV2 microglial cells exposed to 5 μg/mL LTA significantly increased the expression of proinflammatory cytokine, including both TNF-α and IL-6 [35]. At the beginning of this study, to test the cytotoxicity of minocycline in microglia, BV2 and N9 cells were pretreated with various concentrations of minocycline (50–200 μmol/L) for 0.5 h, and then the cells were directly stimulated for 24 h with 5 μg/mL LTA. The cytotoxicity of minocycline combined with LTA in microglia was analyzed using the CCK-8 assay. Minocycline at concentrations ranging from 50 to 200 μmol/L did not significantly reduce the viability of BV2 (Figure 2A) and N9 (Figure 2B) microglial cells. Thus, we selected 50 to 200 μmol/L of minocycline and 5 μg/mL of LTA for the following experiments.

To determine whether minocycline inhibited LTA-induced inflammatory cytokine production in the microglia, saline- or minocycline-pretreated BV2 and N9 cells were stimulated with LTA for 5.5 h. The Western blot results demonstrated that ≥50 of μmol/L minocycline significantly inhibited the TLR2 and inflammatory cytokine expression levels in the BV2 (Figure 2C,E) and N9 (Figure 2D,F) cell lysates. We also confirmed that pretreatment with minocycline significantly reduced the proinflammatory cytokine production of TNF-α and IL-6, but not that of the anti-inflammatory cytokine IL-10, in the supernatants of BV2 (Figure 2G) and N9 cells (Figure 2H). These findings suggest that minocycline attenuates LTA-induced TLR2 signaling pathway activation and proinflammatory cytokine production in microglial cells.

### 3.2. Minocycline Suppresses LTA-Induced STAT3 Phosphorylation and GLS1 Expression in Microglia

**Figure 3:**
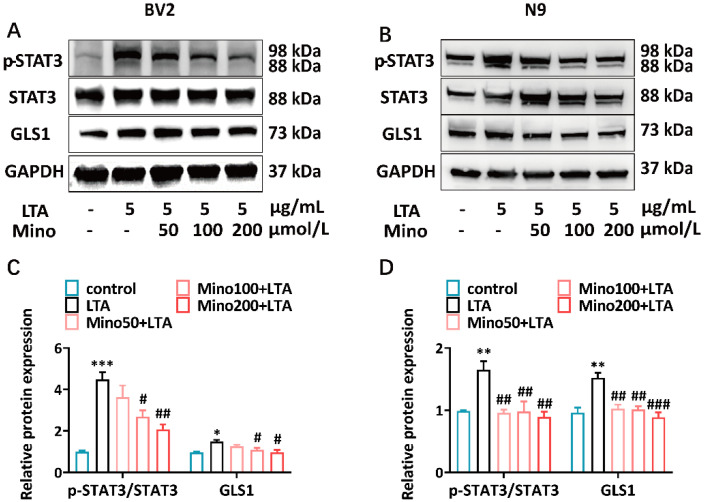
Effects of minocycline on STAT3 activation and GLS1 expression in LTA-induced microglial cells. (**A**,**B**) The expression levels of p-STAT3, STAT3, and GLS1 in the BV2 and N9 cells were analyzed via Western blot analysis. (**C**,**D**) The quantification of the expression levels of p-STAT3/STAT3 and GLS1 in BV2 and N9 cells. The data were normalized according to GAPDH. The mean ± SEM of three separate experiments is represented for all data that have error bars. Compared to the control group, * *p* < 0.05; ** *p* < 0.01; *** *p* < 0.001. Compared to the LTA group, ^#^ *p* < 0.05; ^##^ *p* < 0.01; ^###^ *p* < 0.001. Control, saline-treated group; LTA, cells pretreated with saline and then stimulated with LTA; Mino100 + LTA, cells pretreated with 100 μmol/L minocycline and then stimulated with LTA; Mino200 + LTA, cells pretreated with 200 μmol/L minocycline and then stimulated with LTA.

LTA can activate STAT3 in macrophages and microglial cells [36,37], and activated STAT3 directly binds to the glutaminase (GLS) *GLS1* gene promoter, increasing the expression of GLS1 [38,39]. To examine the influence of minocycline on STAT3 and GLS1, the STAT3 phosphorylation and GLS1 expression levels in the microglia were tested using Western blotting. The results showed that pretreatment with ≥100 μmol/L of minocycline significantly reduced the LTA-induced STAT3 phosphorylation and GLS1 expression in BV2 cells (Figure 3A,C). Similarly, ≥50 μmol/L of minocycline decreased STAT3 phosphorylation and GLS1 expression in N9 cells (Figure 3B,D). These results suggest that minocycline pretreatment may inhibit the LTA-stimulated overexpression of GLS1 by suppressing STAT3 phosphorylation in microglia.

### 3.3. p-STAT3 Inhibition Reduces LTA-Induced Proinflammatory Cytokines and GLS1 Expression in Microglia

**Figure 4:**
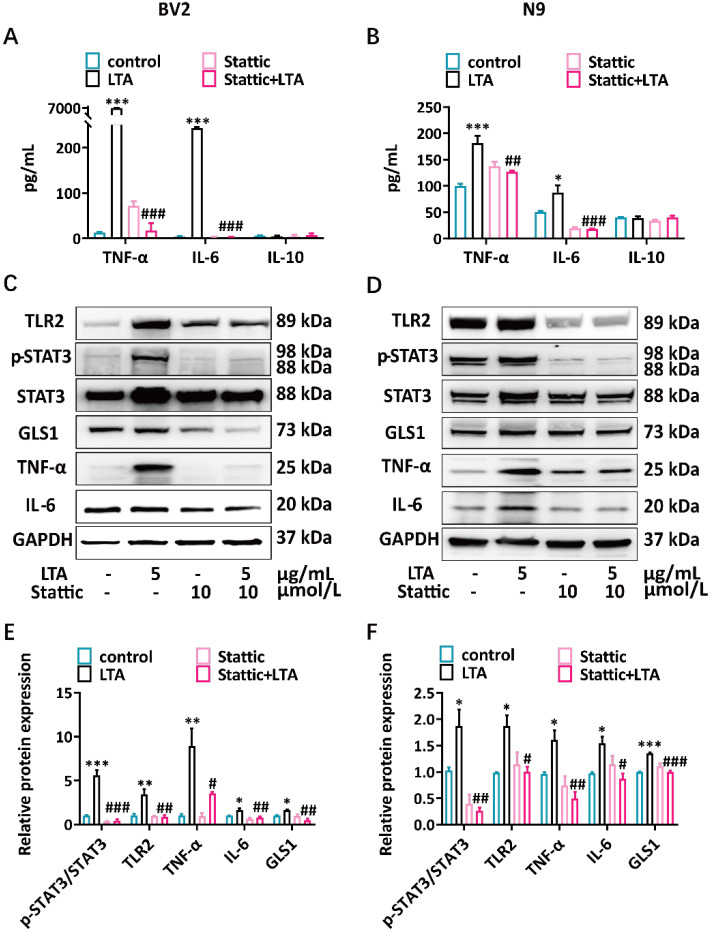
The inhibition of p-STAT3 reduces the proinflammatory cytokines and GLS1 levels in LTA-induced microglial cells. (**A**,**B**) The TNF-α, IL-6, and IL-10 secreted by the BV2 and N9 cells were assessed via ELISA. (**C**,**D**) The expression levels of TLR2, TNF-α, IL-6, GLS1, p-STAT3, and STAT3 in the BV2 and N9 cells were analyzed via Western blot analysis. (**E**,**F**) The quantification of the expression levels of p-STAT3/STAT3, TLR2, TNF-α, IL-6, and GLS1 in the BV2 and N9 cells. The data were normalized according to GAPDH. The mean ± SEM of three separate experiments is represented for all data that have error bars. Compared to the control group, * *p* < 0.05; ** *p* < 0.01; *** *p* < 0.001. Compared to the LTA group, ^#^ *p* < 0.05; ^##^ *p* < 0.01; ^###^ *p* < 0.001. Control, saline-treated group; LTA, cells pretreated with saline and then stimulated with LTA; Stattic, cells pretreated with 10 μmol/L Stattic and then treated with saline; Stattic + LTA, cells pretreated with 10 μmol/L Stattic and then stimulated with LTA.

The nonpeptidic small molecule Stattic specifically inhibits the activity of the STAT3 SH2 domain [40]. We further determined whether the inhibition of STAT3 phosphorylation affected the expression of proinflammatory cytokines and GLS1 in LTA-induced microglial cells. After 0.5 h of saline or Stattic pretreatment, BV2 and N9 cells were directly stimulated with LTA for 5.5 h. The microglial cells’ proinflammatory cytokines TNF-α and IL-6 production were significantly decreased when they were pretreated with Stattic compared to the results for the saline control, while no difference was found for the anti-inflammatory cytokine IL-10 (Figure 4A,B). Furthermore, the Western blot demonstrated that Stattic significantly decreased the phosphorylation of STAT3 and the overexpression of TLR2, TNF-α, and IL-6 stimulated by LTA in the microglial cells (Figure 4C–F). Additionally, we found that Stattic downregulated the GLS1 expression in microglia stimulated with LTA (Figure 4C–F). These data suggest that inhibiting p-STAT3 reduces the levels of LTA-induced proinflammatory cytokines and GLS1 in microglia.

### 3.4. Minocycline Alleviates S. aureus-Induced Neuroinflammation

**Figure 5:**
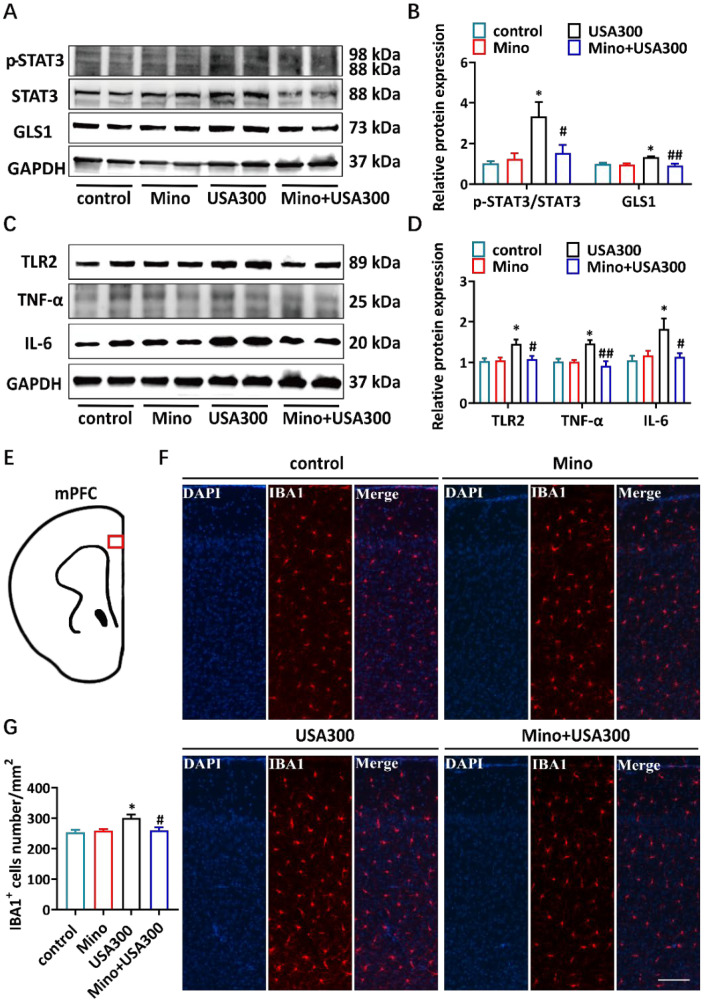
Effects of minocycline on *S. aureus*-induced neuroinflammation in vivo. (**A**) The GLS1, p-STAT3, and STAT3 expression levels in the mPFC of two representative mice were analyzed via Western blot analysis. (**B**) The expression levels of p-STAT3/STAT3 and GLS1 in the mPFC were quantified. The data were normalized according to GAPDH. Data are presented as mean ± SEM, *n* = 4. (**C**) The TLR2, TNF-α, and IL-6 protein expression levels in the mPFC of two representative mice were analyzed via Western blot analysis. (**D**) The expression levels of TLR2, TNF-α, and IL-6 in the mPFC were quantified. The data were normalized according to GAPDH. Data are presented as mean ± SEM, *n* = 4. (**E**) Schematic diagram showing the mPFC area (red box) of the mice analyzed via immunofluorescent staining. (**F**) Representative fluorescence micrographs showing the number of microglia in the mPFC in the various groups (IBA1, red; DAPI, blue); scale bar = 100 μm. (**G**) A quantitative analysis of the number of IBA1^+^ cells in the mPFC. Data are presented as mean ± SEM, *n* = 3. USA300 group compared to the control group, * *p* < 0.05. Mino + USA300 group compared to the USA300 group, ^#^ *p* < 0.05; ^##^ *p* < 0.01. Control, saline-treated group; Mino, mice pretreated with minocycline and then challenged with the saline group; USA300, mice pretreated with saline and then challenged with the USA300 group; Mino + USA300, mice pretreated with minocycline and then challenged with the USA300 group.

We observed that inhibiting the activation of STAT3 could affect the inflammatory response and GLS1 expression in microglial cells. In order to investigate whether minocycline could modify *S. aureus*-stimulated neuroinflammation in vivo, male BALB/C mice (*n* = 7 per group) were intraperitoneally injected with either saline or minocycline (50 mg/kg) daily for 3 days. Half an hour after the last dosage, the mice were intraperitoneally injected with 1 × 10^5^ CFU of *S. aureus*. After 4 days of *S. aureus* infection, the STAT3 phosphorylation and GLS1 expression in the mPFC of the mice were significantly increased in the *S. aureus* infection group when compared to the results for the saline control group (Figure 5A,B). Interestingly, the minocycline significantly reduced the *S. aureus*-induced p-STAT3 and GLS1 expression (Figure 5A,B). These findings indicate that minocycline pretreatment reduces *S. aureus*-induced GLS1 in mice, possibly by regulating the STAT3 signaling pathway.

The inflammatory response in the mPFC tissues in each group was also measured. *S. aureus* infection stimulated TLR2, TNF-α, and IL-6 expression in the mPFC of the mice, whereas minocycline pretreatment significantly reduced these effects (Figure 5C,D). Furthermore, the number of IBA1-labeled microglia was analyzed via immunofluorescent staining in the mPFC of the mice. There were more IBA1-labeled microglia in the *S. aureus*-infected mice than in the saline control group, while the minocycline pretreatment significantly decreased the number of IBA1-labeled microglia (Figure 5E–G). These results suggest that minocycline could attenuate microglia activation and neuroinflammation in the mPFC of *S. aureus*-infected mice, which may lead to the alleviation of behavioral deficits.

### 3.5. Minocycline Ameliorates S. aureus-Induced Anxiety-like Behaviors

**Figure 6:**
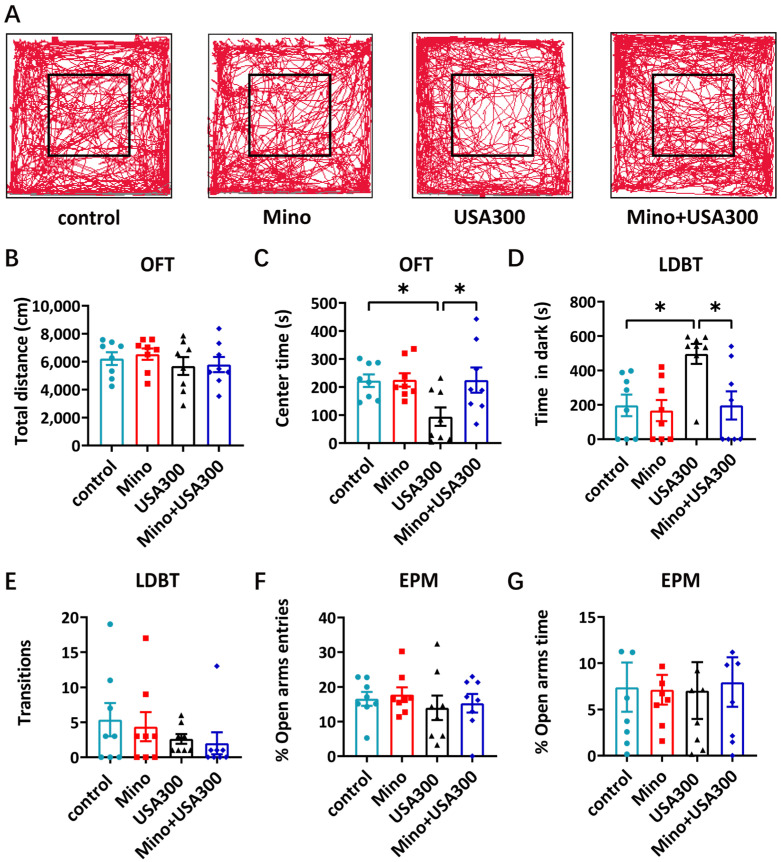
Minocycline alleviates anxiety-like behaviors in *S. aureus*-infected mice. (**A**) Representative tracks of mice in different groups in the OFT. (**B**) The total distance and (**C**) the time spent in the central area by the mice in the open field test. (**D**) The amount of time that the mice remained in the dark compartment and (**E**) the number of times that they switched between the light and dark compartments in the light–dark box test. (**F**) The percentage of open arm entries and (**G**) the proportion of time spent in the elevated plus maze did not differ significantly. (each group, *n* = 8). The mean ± SEM is used to represent all data; * *p* < 0.05. Control, saline-treated group; Mino, mice pretreated with minocycline and then challenged with the saline group; USA300, mice pretreated with saline and then challenged with the USA300 group; Mino + USA300, mice pretreated with minocycline and then challenged with the USA300 group.

Neuroinflammation partially contributes to behavioral deficits [41]. Our earlier research demonstrated that infection with *S. aureus* caused neuroinflammation and anxiety-like behaviors in mice [31]. Therefore, we aimed to determine whether minocycline could ameliorate anxiety-like behaviors caused by *S. aureus* infection. The animal behavioral experimental design is shown in Figure 1. The mouse survival rates were 100% (Figure S1), and their body weights did not decrease (Tabel S1) within 7 days of experiment. The total distance traveled over 30 min did not differ in the open field test (OFT) (Figure 6A,B). Compared to the saline control group, the *S. aureus*-infected mice spent significantly less time in the central area (Figure 6A,C), while minocycline pretreatment reversed the decreased time spent in the center area (Figure 6A,C).

Furthermore, minocycline ameliorated the anxiety-like behavior caused by *S. aureus*-infection, as confirmed by the light–dark box test (LDBT). Compared to the saline control group, the *S. aureus*-infected mice spent significantly more time in the dark box (Figure 6D). Minocycline pretreatment reduced the time spent by the *S. aureus*-infected mice in the dark box compared to the results for the single *S. aureus*-infected mice (Figure 6D). However, the number of transitions between the dark and light boxes (Figure 6E), the percentage of open arm entries (Figure 6F), and the percentage of time spent in the open arms did not differ in each group in the elevated plus maze (EPM) test (Figure 6G). These behavioral testing results differed from the typical LPS-induced anxiety-like behaviors, caused by the complex components of *S. aureus*. Collectively, these findings indicate that minocycline pretreatment was able to effectively alleviate anxiety-like behaviors induced by *S. aureus* infection in a mouse model.

## 4. Discussion

*S. aureus* produces numerous virulent toxins to trigger different inflammatory responses during infection [42,43]. Minocycline has been reported to have a therapeutic effect on infection-induced behavior [17,23,26,44]. However, the protection afforded by minocycline to ameliorate *S. aureus*-induced anxiety-like behavior is rarely reported. In this study, we first found that minocycline ameliorated the anxiety-like behavior caused by *S. aureus* infection. Microglia are highly specialized macrophages that are found in the brain [45]. TLR2, which is present in microglia, plays a crucial role in sensing PAMPs derived from *S. aureus*, including lipoproteins, LTA, and PGN [46,47,48,49]. As an analog of LPS from Gram-negative bacteria, LTA is a surface-associated adhesion amphiphile derived from Gram-positive bacteria that can be recognized by TLR2, thereby initiating immune responses [50,51,52,53]. Minocycline reduced LPS-induced TLR2 surface expression on brain microglia [23]. LTA can cross the BBB to activate microglia and stimulate the release of proinflammatory cytokines [54,55]. We found that *S. aureus* LTA induced high TLR2 expression and proinflammatory cytokine production in microglial cells, and minocycline strongly suppressed this effect (Figure 2C,D). Minocycline may affect the production of proinflammatory cytokines by directly inhibiting LTA-induced TLR2 expression.

STAT3 is a member of the STAT protein family and functions as a transcription factor to regulate cytokine signaling, which is activated by Janus kinases (JAK) [56]. The activation of the microglia STAT3 pathway mediates the neuroinflammation [57,58]. The activation of microglia has been reported to be linked to behavioral changes in sepsis patients [59]. A novel lipophilic compound reduces the inflammatory response of LPS-stimulated microglia and exhibits a neuroprotective effect by inhibiting STAT3 phosphorylation [60]. LTA combines with PGN to trigger an inflammatory response and to activate the JAK/STAT pathway [61]. We found that STAT3 phosphorylation was upregulated in LTA-stimulated microglia (Figure 3A,B). Minocycline inhibited the activation of STAT3 induced by LPS, exerting anti-inflammatory and neuroprotective effects [26]. According to our findings, minocycline also inhibited STAT3 phosphorylation and reduced the TNF-α and IL-6 production in LTA-stimulated microglia (Figure 4C–F). Our study provided evidence that minocycline can modulate the phosphorylation of STAT3 in microglia.

GLS has two isoforms, GLS1 and GLS2, which regulate microglia activation and neuroinflammation [27,62]. As a transcription factor, activated STAT3 directly increases the production of GLS1 [27,38]. High GLS1 protein levels have been found in many neurological and psychiatric diseases [63,64]. In this study, minocycline effectively reduced *S. aureus* infection-induced high GLS1 expression in the mouse mPFC (Figure 5A,B). It is possible that minocycline reduces the GLS1 expression in the mPFC of mice by downregulating STAT3 phosphorylation. The pathogenesis of the anxiety-like behaviors caused by *S. aureus* infection depends on the neuroinflammation mediated by microglia [31,65]. Minocycline reduced STAT3 phosphorylation and neuroinflammation in the mPFC of *S. aureus*-infected mice (Figure 5A,C). Our preliminary data suggested that minocycline ameliorated *S. aureus* infection-induced anxiety-like behavior (Figure 6). Overall, our results suggest that minocycline plays a protective role regarding anxiety-like behavior during *S. aureus* infection, and these findings require further investigation.

Nevertheless, our study is subject to limitations. First, in Alzheimer’s disease (AD), Parkinson’s disease (PD), and Huntington’s disease (HD) models, activation of the STAT3 pathway in astrocytes has been observed [66]. LPS evokes proinflammatory cytokine levels in astroglia through STAT3 phosphorylation [67]. The effect of *S. aureus* infection on STAT3 in astrocytes is not explored here, which is a limitation of this study. We speculate that astrocytes and microglia are likely to play a synergistic role in inducing neuroinflammation during the process of *S. aureus* infection. Therefore, the potential impact of astrocyte-linked mechanisms on *S. aureus*-induced neuroinflammatory responses (directly and/or indirectly) needs to be addressed in a future study. Second, minocycline ameliorated *S. aureus* infection-induced neuroinflammation in the mPFC and anxiety-like behaviors in mice. We are not ruling out the possibility that other brain regions may underlie the functions of minocycline. Third, anxiety-like behaviors induced by *S. aureus* infection differ from the typical anxiety-like behaviors induced by LPS, which could be due to the complex components of *S. aureus*.

## 5. Conclusions

In conclusion, our study demonstrates that minocycline alleviates *S. aureus* infection-induced neuroinflammation and anxiety-like behaviors by suppressing the TLR2 and STAT3 signaling pathways in microglia. Minocycline supplementation may represent a potential strategy for the treatment of anxiety disorders caused by *S. aureus* infection; this is worthy of further validation through a series of clinical and basic studies.

## Data Availability

The data supporting the conclusions of this article are available from the corresponding author upon request. The data are not publicly available due to specific ethical and privacy considerations.

## Supplementary Materials

The following supporting information can be downloaded at: https://www.mdpi.com/article/10.3390/brainsci15020128/s1, Figure S1: Effects of USA300 infection and minocycline pretreatment on the weight of mice; Table S1: Effects of USA300 infection and minocycline pretreatment on the percent survival of mice.
