# Supplementary material for: Minocycline Ameliorates Staphylococcus aureus-Induced Neuroinflammation and Anxiety-like Behaviors by Regulating the TLR2 and STAT3 Pathways in Microglia
Source: Brain Sci. 2025 Jan 28;15(2):128. doi: 10.3390/brainsci15020128 (PMC11853265; doi:10.3390/brainsci15020128)
Supplement: Supplementary file 1 [file brainsci-15-00128-s001.zip › brainsci-3440783-supplementary.pdf]

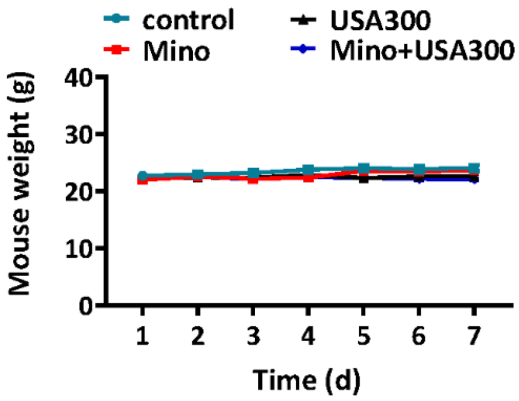


**Figure S1. Effects of USA300 infection and minocycline pretreatment on the weight of mice.** Variation of mouse weights during treatment (n = 8). control, saline-treated group; Mino, mice pretreated with minocycline and then challenged with the saline group; USA300, mice pretreated with saline and then challenged with the USA300 group; Mino+USA300, mice pretreated with minocycline and then challenged with the USA300 group.

**Table S1. Effects of USA300 infection and minocycline pretreatment on the percent survival of mice.**

| **Time (d)** | **Survival rates of mice (%)** | | | |
|---|---|---|---|---|
| | **control** | **Mino** | **USA300** | **Mino+USA300** |
| **1** | 100 | 100 | 100 | 100 |
| **2** | 100 | 100 | 100 | 100 |
| **3** | 100 | 100 | 100 | 100 |
| **4** | 100 | 100 | 100 | 100 |
| **5** | 100 | 100 | 100 | 100 |
| **6** | 100 | 100 | 100 | 100 |
| **7** | 100 | 100 | 100 | 100 |

control, saline-treated group; Mino, mice pretreated with minocycline and then challenged with the saline group; USA300, mice pretreated with saline and then challenged with the USA300 group; Mino+USA300, mice pretreated with minocycline and then challenged with the USA300 group.
